# Presence of Spodoptera frugiperda Multiple Nucleopolyhedrovirus (SfMNPV) Occlusion Bodies in Maize Field Soils of Mesoamerica

**DOI:** 10.3390/insects14010080

**Published:** 2023-01-13

**Authors:** Trevor Williams, Guadalupe del Carmen Melo-Molina, Jaime A. Jiménez-Fernández, Holger Weissenberger, Juan S. Gómez-Díaz, Laura Navarro-de-la-Fuente, Andrew R. Richards

**Affiliations:** 1 Instituto de Ecología AC (INECOL), Xalapa 91073, Veracruz, Mexico; 2 El Colegio de la Frontera Sur (ECOSUR), Tapachula 30700, Chiapas, Mexico; 3 El Colegio de la Frontera Sur (ECOSUR), Chetumal 77014, Quintana Roo, Mexico; 4 CSIRO Division of Entomology, Canberra, ACT 2601, Australia

**Keywords:** Baculoviridae, *Alphabaculovirus*, virus persistence, insect bioassay, genetic diversity, soil type, fall armyworm

## Abstract

**Simple Summary:**

Nucleopolyhedroviruses of caterpillars (Lepidoptera) produce occlusion bodies (OBs) that protect the virions within a protein matrix. This allows the virus to persist in the environment until ingested by a susceptible insect. The soil is an important reservoir of OBs that can be transported to plants for transmission to the host caterpillar. The Spodoptera frugiperda multiple nucleopolyhedrovirus is a lethal pathogen of the fall armyworm, a major pest of maize, rice, and sorghum. Analysis of 186 soil samples collected from maize fields in southern Mexico, Belize, and Guatemala revealed that almost 19% of samples had OBs present at low concentrations. Genetic analysis revealed the presence of genetic diversity in the soil OB populations. The presence of OBs was higher in maize fields with living crops and in specific types of soils. These findings suggest that the soil could be a valuable source of genetic diversity for the design of virus-based insecticides to control this important pest.

**Abstract:**

The occlusion bodies (OBs) of lepidopteran nucleopolyhedroviruses can persist in soil for extended periods before being transported back on to the foliage for transmission to the host insect. A sensitive insect bioassay technique was used to detect OBs of Spodoptera frugiperda multiple nucleopolyhedrovirus (SfMNPV) in 186 soil samples collected from maize fields in the southern Mexican states of Chiapas, Tabasco, Campeche, Yucatán, and Quintana Roo, as well Belize and Guatemala. Overall, 35 (18.8%) samples proved positive for SfMNPV OBs. The frequency of OB-positive samples varied significantly among Mexican states and countries (*p* < 0.05). Between 1.7 and 4.4% of *S. frugiperda* larvae that consumed OB-positive samples died from polyhedrosis disease. Restriction endonuclease analysis using PstI and HindIII confirmed that the soil-derived isolates were strains of SfMNPV and that genetic diversity was evident among the isolates. The prevalence of OB-positive soil samples did not differ with altitude or extension (area) of the maize field, but it was significantly higher in fields with the presence of living maize plants compared to those containing dead plants or crop residues (*p* < 0.05). Georeferenced soil samples were used to identify soil types on digitized soil maps. Lithosol and Luvisol soils had a higher than average prevalence of OB-positive samples (42–45% positive) (*p* = 0.006), as did Andosol, Gleysol, and Vertisol soils (33–60% OB-positive), although the sample sizes were small (<5 samples) for the latter three soils. In contrast, Cambisol soils had a lower than average prevalence of OB-positive samples (5% positive). Bioassays on Acrisol, Fluvisol, Phaeozem, and Rendzina soils resulted in intermediate levels of OB-positive samples. We conclude that certain soil types may favor OB persistence and virus-mediated biological pest control. The soil is also likely to provide a valuable source of genetic diversity for the design of virus-based insecticides against this pest.

## 1. Introduction

Nucleopolyhedrovirus occlusion bodies (OBs) that are released from virus-killed insects are washed off the plant by the action of rainfall or by the senescence and shedding of OB-contaminated leaves and subsequently enter the soil [1,2]. As a result, the soil represents an important environmental reservoir of nucleopolyhedrovirus OBs [3], although the persistence of OB populations in soil is poorly understood [4]. The OB structure allows the occlusion-derived virions (ODVs) to remain viable for months or years in soil, given favorable conditions of pH and temperature [5]. OBs in soil are also protected from the adverse effects of solar ultraviolet (UV) radiation that can rapidly inactivate these viruses [6]. OBs in the soil are transported back on to host plants during plant growth through the soil, through the action of wind-blown dust and rain-splash [7,8,9], or they are carried on the bodies of foraging invertebrates [10].

As OBs adhere strongly to soil particles, it is difficult to separate them by conventional techniques [11]. Techniques have been developed to detect the presence of OBs in soil by polymerase chain reaction (PCR), although amplification can be hindered by a diversity of soil components [12,13,14]. A sensitive bioassay technique has been developed involving feeding early instar larvae with mixtures of soil and semi-synthetic diet [15]. This technique has been used to obtain novel occluded virus isolates [16] and to study the abundance and genetic composition of soil OB populations [17,18] and their interaction with soil-dwelling invertebrates [19,20].

The Spodoptera frugiperda multiple nucleopolyhedrovirus (SfMNPV) (family *Baculoviridae*; genus *Alphabaculovirus*) is a common pathogen in natural populations of the fall armyworm, *Spodoptera frugiperda* (Lepidoptera: Noctuidae) in the Americas, and it has now been reported in China [21], India [22], and Indonesia [23], where the pest has recently invaded. The virus usually causes mortality of between 1% and 5% in *S. frugiperda* larvae [24,25,26], although some populations can experience seasonal epizootics [27,28]. This virus has attracted attention as the active ingredient in the development of biological insecticides against this pest, and several commercial products have been developed [29].

As part of a long-term study on the biology, ecology, and bioinsecticide potential of the *S. frugiperda*—SfMNPV pathosystem, we examined the prevalence of SfMNPV OBs in maize field soils in southern Mexico and part of Central America, over an area of >2000 km^2^. The sampling program generated multiple isolates, some of which were gifted to other researchers who examined their insecticidal characteristics [30]. As digitalized maps of soil types in this region recently became available, we used our data, collected two decades ago, to analyze the relationship between soil type and the presence of SfMNPV OBs in maize fields in the region.

## 2. Materials and Methods

### 2.1. Insect Colonies and Reference Virus Isolate

A laboratory colony was started in 1996 using larvae of *S. frugiperda* collected from maize fields close to the city of Tapachula, Chiapas, Mexico. Larvae were reared individually in 30 mL plastic cups with a piece of semi-synthetic diet based on soya, yeast, and maize flour without formaldehyde [31]. For oviposition, groups of adults were placed in paper bags with a plastic dish containing a cotton pad soaked in 10% honey solution and maintained at 25–28 °C. This colony was used for the isolation of SfMNPV OBs from soil samples. In the year 2000, a sample from this colony was sent to the Universidad Pública de Navarra, Spain, reared, and analyzed by reverse transcription PCR using primers targeting the *ie-0* and *polh* genes and was found to be free of sublethal nucleopolyhedrovirus infection [32].

A second colony of *S. frugiperda* was started in 2021 using larvae collected from maize plants in a field close to Cempoala, Veracruz. This colony was treated identically to that of the first colony except that it was maintained at a constant 26 ± 1 °C in a temperature-controlled room. This colony was used to amplify isolates prior to restriction endonuclease analysis. PCR analysis using primers targeted at the *sf58* gene [33] or the *polh* gene [34] did not result in amplification of DNA samples taken from larvae, pupae, or adults, suggesting that this colony did not harbor sublethal nucleopolyhedrovirus disease.

The Nicaraguan isolate of SfMNPV (SfMNPV-NIC) was used as a reference isolate that has been characterized in detail in previous studies [35,36,37]. This isolate naturally comprises nine genotypic variants that are present in varying proportions.

### 2.2. Soil Sampling

Between 30 June and 20 October 2000, a total of 186 soil samples were collected from maize fields in the southern Mexican states of Chiapas (120 samples), Tabasco (1 sample), Campeche (8 samples), Yucatán (16 samples), and Quintana Roo (10 samples), as well as parts of Belize (4 samples) and Guatemala (27 samples). Only a single sample was taken in Tabasco as the road to Campeche passed through this state for only a very short distance (~5 km).

Sampling was non-systematic and consisted of driving along roads and selecting accessible fields with evidence of maize cultivation (planted crop or crop residues). In areas of intense maize production, fields were sampled at intervals of hundreds of meters (shortest distance 100 m) or a few kilometers, whereas in other areas, such as Belize where the dominant crop is sugarcane, maize fields were sampled at intervals of tens of kilometers (longest distance ~80 km). The aim was to obtain a range of samples in each area that we passed through. A soil sample was taken by scraping away the upper 5 cm of soil and collecting ~100 g of soil at a depth of 5–10 cm at five random points in each field to produce a sample of ~500 g that was placed in a black plastic bag, mixed by shaking, sealed, and labeled with a site code. Following the procedures of Richards & Christian [15], the uppermost 5 cm of soil was not sampled as OBs in this layer would be exposed to extremes of solar UV radiation and high temperatures that may have affected their viability.

The geographical coordinates and altitude of each field were recorded using a GPS locator (Garmin III Plus, Garmin International Inc., Olathe, KS, USA); the area of the field was estimated (based on the approximate dimensions and shape of the field); the state of the crop was classified in terms of growth status (living or dead), as maize is commonly folded over and allowed to dry prior to harvesting in this region, or the presence of plant residues from a previously harvested crop (Supplemental File S1). Soil samples were taken to the laboratory in ECOSUR, Tapachula, Chiapas, spread on sheets of paper, and allowed to air dry for 2–5 days in a dark room at 26–28 °C and then prepared for insect bioassay. Following the bioassay procedure, all soils were autoclaved prior to disposal to eliminate soil-borne pathogens.

### 2.3. Bioassay of Soil Samples

The soil bioassay procedure involves larvae that are fed mixtures of soil and semi-synthetic diet [15]. Air-dried soil samples were passed through a 1 mm sieve to eliminate stones and large pieces of organic material. Sieves were washed and decontaminated by treatment with 0.5% sodium hypochlorite solution to avoid cross-contamination. A 10 g sample of sieved soil was placed in a 250 mL plastic cup and mixed thoroughly with 90 g of semi-synthetic diet using a disposable wooden spatula to produce a smooth uniform paste. The soil–diet mixture was then distributed evenly among 30 plastic cups (30 mL capacity), and two *S. frugiperda* first instars from the first laboratory colony were placed in each cup. An additional group of 10 larvae per sample was reared individually with a mixture of sterilized soil and diet as controls. Larvae were incubated at 25 °C and were checked at 48 h intervals until death or pupation. A small portion of each dead larvae was taken with a toothpick, smeared on a microscope slide, stained using Giemsa stain, and examined for the presence of OBs under a phase-contrast microscope at ×1000 with oil immersion [38]. The efficacy of the soil sterilization procedure was checked by autoclaving samples of 10 g soil that had been spiked with 1.9 × 10^6^ OB/g soil (equivalent to the estimated LC_50_ concentration mentioned in Section 3.1). None of the larvae that were treated with soil+diet mixtures of autoclaved soil succumbed to polyhedrosis disease (3 replicates of 24 larvae each).

OB-positive larva were macerated in 50 µL of distilled water, mixed with 10% sucrose and 0.05% Fluorella blue, and used to inoculate fourth instar larvae that had been starved overnight. Larvae that consumed the inoculum within 15 min were individualized in the wells of a 24-well cell culture plate with a piece of diet. Larvae were incubated at 25 ± 1 °C and were checked daily for signs of polyhedrosis disease until death. Virus-killed larvae were placed in 1.5 mL centrifuge tubes and stored at −80 °C for 21 years. Some of the samples were gifted to other researchers to perform comparative studies on the insecticidal characteristics of the isolates.

A selection of 32 isolates was subjected to PCR amplification using primers Sf58.1 (5’-GTCCTCGGTGCTGAATCAGG-3′) and Sf58.2 (5′-TTACGTAGGTGCTGGAGGAG-3′) targeted at the *sf58* gene of SfMNPV [33]. For this, viral DNA was extracted from OBs as follows. Each infected larva was homogenized, filtered through an 80 micron pore steel mesh, and centrifuged at 2500 rpm for 5 min to pellet debris. The OB suspension was placed on a cushion of 50% glycerol and centrifuged at 12,000 rpm for 10 min. The resulting OB pellet was washed with distilled water and resuspended in 200 µL of distilled water. A 100 µL volume of 3xDAS buffer (0.3 M Na_2_CO_3_, 0.5 M NaCl, 0.03 M EDTA; pH 10.5) was added to the OB suspension and incubated at 45 °C for 45 min to release occlusion-derived virions (ODVs). The suspension was centrifuged at 3000 rpm for 5 min to remove undissolved OBs, and the supernatant was centrifuged at 12000 rpm for 10 min. The resulting pellet of ODVs was resuspended in 100 µL MilliQ water, 10 µL of 10% SDS, and 3 µL of proteinase K (20 mg/mL), followed by incubation at 45 °C for 45 min. Viral genomic DNA was then extracted by treatment with phenol-chloroform and precipitated in absolute ethanol with 1/10 vol 3 M sodium acetate at −20 °C overnight. The DNA pellet was washed with 70% ethanol and resuspended in 50 µL MilliQ water, and the concentration was measured in a UV-spectrometer at 260 nm (BioSpec-Nano, Shimadzu, Japan).

A 1 µL sample of the resulting DNA was amplified at 95 °C for 3 min, followed by 35 cycles of 95 °C for 30 s, 59 °C for 30 s, 72 °C for 1 min, and final extension at 72 °C for 7 min. Electrophoresis was performed in 1.2% agarose in TBE buffer (100 mM Tris, 90 mM boric acid, 1 mM EDTA; pH 8.3) containing 35 µL/liter of ethidium bromide (10 mg/mL) and photographed on a transilluminator (UV ChemiDoc XRS+ System with Image Lab Software; Bio-Rad, Hercules, CA, USA). A sample of SfMNPV-NIC (10^6^ OBs) was included as a positive control and water as a negative extraction control.

The frequency of OB-positive soil samples was compared across the states of southern Mexico and Belize and Guatemala by Fisher’s exact test, whereas the prevalence of virus-induced disease in bioassay larvae exposed to soil samples was compared across states and countries by fitting a generalized linear model with a binomial error structure in GLIM 4 [39].

To quantify the sensitivity of the soil bioassay, the procedure was performed using a characterized clay soil (pH 5.2) from the grounds of the Instituto de Ecología AC, Xalapa, Mexico, which was studied previously [20]. For this, 1 mL suspensions of SfMNPV-NIC OBs were mixed with 10 g soil samples to produce concentrations of 2 × 10^4^, 2 × 10^5^, 2 × 10^6^, 2 × 10^7^ OBs/g soil. Control samples were treated with 1 mL distilled water. Each OB-contaminated soil was mixed with 90 g semi-synthetic diet and distributed among the wells of 24 well cell culture plates, and a single *S. frugiperda* first instar larva from the Cempoala colony was placed in each well and incubated at 27 ± 1 °C in darkness. Larvae that died from polyhedrosis disease (confirmed by examination of Giemsa-stained smears) were counted and recorded. The procedure was replicated four times using different batches of insects. The results were subjected to logit regression in GLIM 4 [39].

### 2.4. Restriction Endonuclease Analysis of Soil Isolates

To examine the presence of genetic diversity in soil isolates, eight isolates were selected at random. These isolates were amplified in *S. frugiperda* fourth instars from the Cempoala colony. Larvae were individualized, starved overnight, and then allowed to drink a suspension of 10^8^ OBs/mL in 10% sucrose solution with 0.05% Fluorella blue. Larvae that consumed the inoculum within 15 min were individualized in the wells of a 24-well cell culture plate with a piece of diet. Larvae were incubated at 27 ± 1 °C and were checked daily for signs of polyhedrosis disease. Virus-killed larvae were placed in 1.5 mL centrifuge tubes and stored at −20 °C.

To obtain viral DNA, larvae were individually homogenized in 1 mL distilled water. OBs were then purified, and DNA was extracted, purified by phenol-chloroform treatment, and resuspended in 100 µL of MilliQ water as described in Section 2.3. Samples of 1 µg DNA of each isolate were digested with restriction endonucleases PstI or HindIII (New England Biolabs, Ipswich, MA, USA) at 37 °C following the manufacturer’s recommendations. After 6 h, the reaction was stopped by addition of loading buffer, and samples were loaded in a 0.6% (PstI) or 0.8% (HindIII) agarose gel in TBE buffer containing 35 µL/liter of ethidium bromide (10 mg/mL). Electrophoresis was performed overnight at 30 V. Each gel was then photographed on a transilluminator (UV ChemiDoc XRS+ System with Image Lab Software; Bio-Rad, Hercules, CA, USA).

### 2.5. Relationship between Soil OBs and Environmental Factors

Data collected on the altitude (GPS data) and the estimated size of maize fields were compared for OB-positive and OB-negative soil samples by Kruskal–Wallis test and were reported as medians ± interquartile range (IQR). The stage of the crop cycle was classified as (i) green living stages from seedling to maize cob production or (ii) dry, dead plants and crop residues, as farmers twist and bend the maize stem to allow the cobs to dry in the field before they are harvested. The frequency of positive and negative samples was compared for each crop stage by contingency table analysis.

The GPS-referenced location of each sample was plotted on digitized maps produced using vector data on southern Mexico [40] or Belize and Guatemala produced by the Food and Agriculture Organization of the United Nations [41]. Maps were drawn, and sample points were plotted using ArcGIS 10.4 software [42]. Intensive sampling was performed in the southernmost Soconusco region of Chiapas State, so this region was plotted on a separate map for clarity. Mapped information was used to identify soil type and subtype for each of the 186 samples analyzed. The prevalence of OB-positive samples was compared among soil types by contingency table analysis. Soils with fewer than five samples were excluded from this analysis.

## 3. Results

### 3.1. Calibration of the Soil Bioassay

Mortality in the soil bioassay ranged from 1% in the 2 × 10^4^ OB/g soil concentration to 99% in the 2 × 10^7^ OB/g soil concentration (*χ*^2^ = 310.2, *df* = 1, *p* < 0.001). No virus-induced deaths were observed in the control. The slope (± SE) of the logit regression was 1.383 ± 0.153, and the estimated LC_50_ was 1.9 × 10^6^ OBs/g soil (range of 95% C.I.: 1.48 × 10^6^–2.48 × 10^6^). There was no evidence of overdispersion in the data (residual deviance/residual d.f. = 1.02).

### 3.2. Bioassay of Soil Samples from Maize Fields

**Figure 1:**
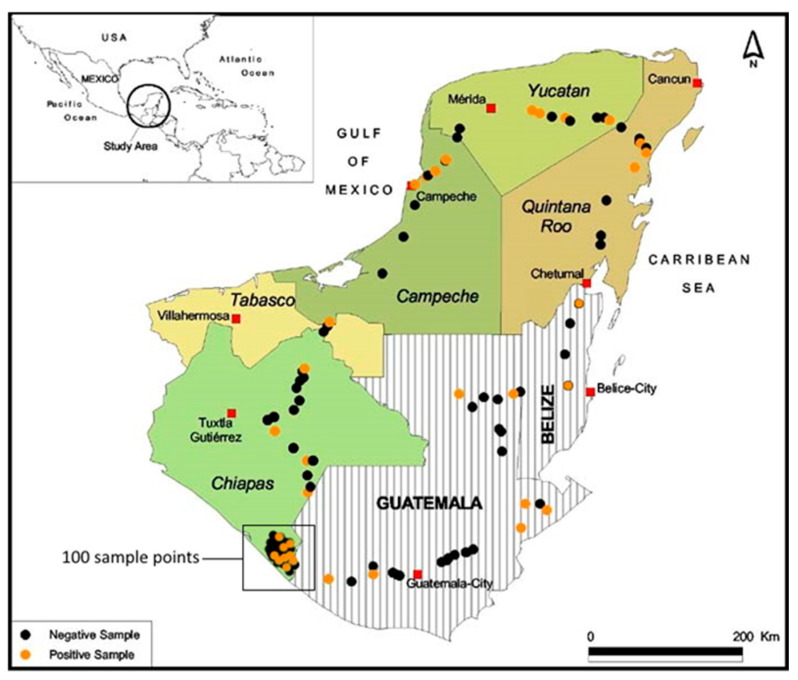
Distribution of soils samples that proved positive (orange points) or negative (black points) for SfMNPV OBs in the soil-diet bioassay. Samples were distributed across fives states of southern Mexico, Belize, and Guatemala. Intensive sampling was performed in the Soconusco region in the south of Chiapas State, Mexico (shown enclosed in a black rectangle).

Of the 186 soil samples that were collected from maize fields, 35 (18.8%) proved positive for SfMNPV OBs by insect bioassay (Figure 1, Table 1). The prevalence of OB-positive samples varied significantly by state and country (Fisher’s exact *p* = 0.003). A single negative sample from Tabasco state was not included in the analysis. The highest number of OB-positive samples (N = 14) was obtained from Chiapas state, reflecting the intensive sampling in this state. The remaining states in Mexico had intermediate numbers of OB-positive samples (N = 3–6). Two positive samples were obtained from Belize, and six positive samples were collected in Guatemala (Table 1).

On average (±SE), 1.74 ± 0.18 larvae died from polyhedrosis disease in the OB-positive samples (range 1–4 larvae/sample). Lethal polyhedrosis disease was confirmed by examination of Geimsa smears. The prevalence of infection (range 1.7–4.4%; Table 1) did not differ significantly among samples from different states and countries (GLM *χ*^2^ = 6.245, *df* = 5, *p* = 0.283). An average (±SE) of 2.14 ± 0.38 larvae (range 0–8) died from other causes in OB-positive samples, mainly bacterial deaths, and these larvae were not considered further. None of the control larvae that consumed sterilized soil samples succumbed to polyhedrosis disease.

All 32 isolates tested proved positive for PCR amplification of the *sf58* gene, resulting in the expected 306 bp product, although there appeared to be slight variation in the amplicon size, suggesting the presence of genotypic variation among isolates (Supplemental Figure S3).

### 3.3. Restriction Endonuclease Analysis of Soil Isolates

**Figure 2:**
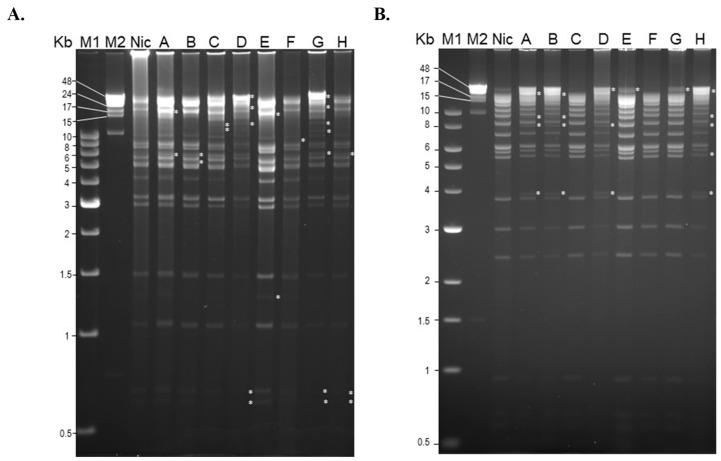
Restriction endonuclease analysis of a selection of soil-derived isolates of SfMNPV (lanes A-H) compared to the SfMNPV-NIC reference isolate (Nic). Genomic DNA was digested with (**A**) PstI or (**B**) HindIII and subjected to electrophoresis in 0.6% and 0.8% agarose, respectively. The isolates used for this analysis were SfMNPV-NIC (labeled Nic) sample #121 (labeled A), #36 (B), #37 (C), #19 (D), #152 (E), #92 (F), #177 (G), and #50 (H), following the sample numbers given in Supplemental File S1. Restriction fragments and sub-molar bands that differed from those of SfMNPV-NIC are labeled with an asterisk. Molecular markers were NEB 1 Kb ladder (M1) and NEB λ mono-cut DNA (M2).

Restriction endonuclease analysis of eight randomly selected isolates indicated considerable levels of genetic diversity across these isolates. Compared to the SfMNPV-NIC reference isolate, all the soil-derived isolates differed in the number and size of restriction fragments and also in the presence of sub-molar fragments that suggest that individual isolates likely comprise mixtures of genotypes (clear differences are marked with asterisks in Figure 2A,B). Digestion with PstI and HindIII revealed polymorphism that was apparent across the range of fragment lengths. The results of these analyses should be viewed with caution as the largest fragments (>48 Kb) may be difficult to distinguish from undigested genomic DNA, or partially digested DNA in the case of some sub-molar bands. The three short fragments of between 0.5 and 1 Kb produced by HindIII treatment were similar across all isolates, but they are not easily visualized in Figure 2B.

### 3.4. Relationship between Soil OBs and Environmental Factors

Maize fields were sampled at altitudes between 8 and 2630 m above mean sea level, but altitude did not differ significantly between OB-positive soil samples (median [IQR]: 57 m [105]) and OB-negative samples (69 m [197]) (Kruskal–Wallis *H* = 1.039, *df* = 1, *p* = 0.308). Maize fields varied in estimated area from 0.04 to 15 ha, but the size the field did not differ significantly between OB-positive soil samples (median [IQR]: 1 ha [2.5]) and OB-negative samples (1 ha [1.25]) (Kruskal–Wallis *H* = 0.00, *df* = 1, *p* = 0.997).

**Figure 3:**
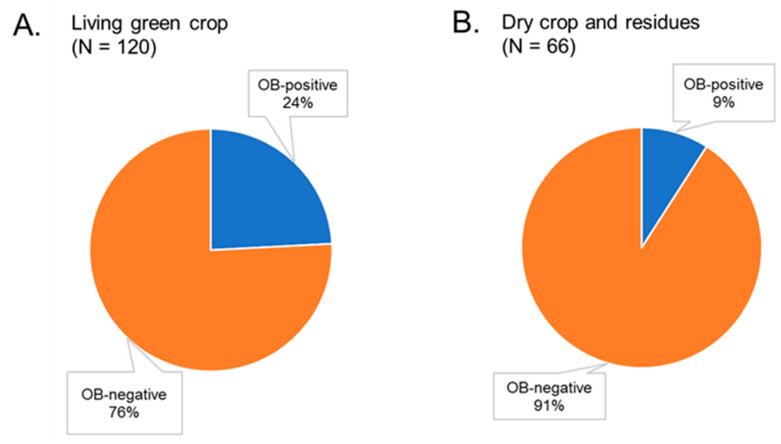
Prevalence of OB-positive and -negative samples taken from maize fields with (**A**) living green crops and (**B**) dry crops or post-harvest crop residues. Percentage values shown in gray rectangles are based on the indicated sample sizes (N).

The presence of the crop in the green living stages (from seedling to cob production) was associated with an increased probability of OB-positive soil samples (Figure 3A,B). Overall, 29 out of 120 (24%) maize fields with living crops had OB-positive soils, whereas only 6 out of 66 (9%) maize fields with dead, dry crops and crop residues proved OB-positive in the insect bioassay (*χ*^2^ = 6.335, *df* = 1, *p* = 0.012). Looking at these results in another way, 83% (29 out of 35) of OB-positive soils originated from fields with green living crops, whereas only 17% (6 out of 35) of the OB-positive soils originated from fields with dry crops and crop residues. In this region, farmers twist and fold the maize stem to allow the cobs to dry before harvesting.

**Figure 4:**
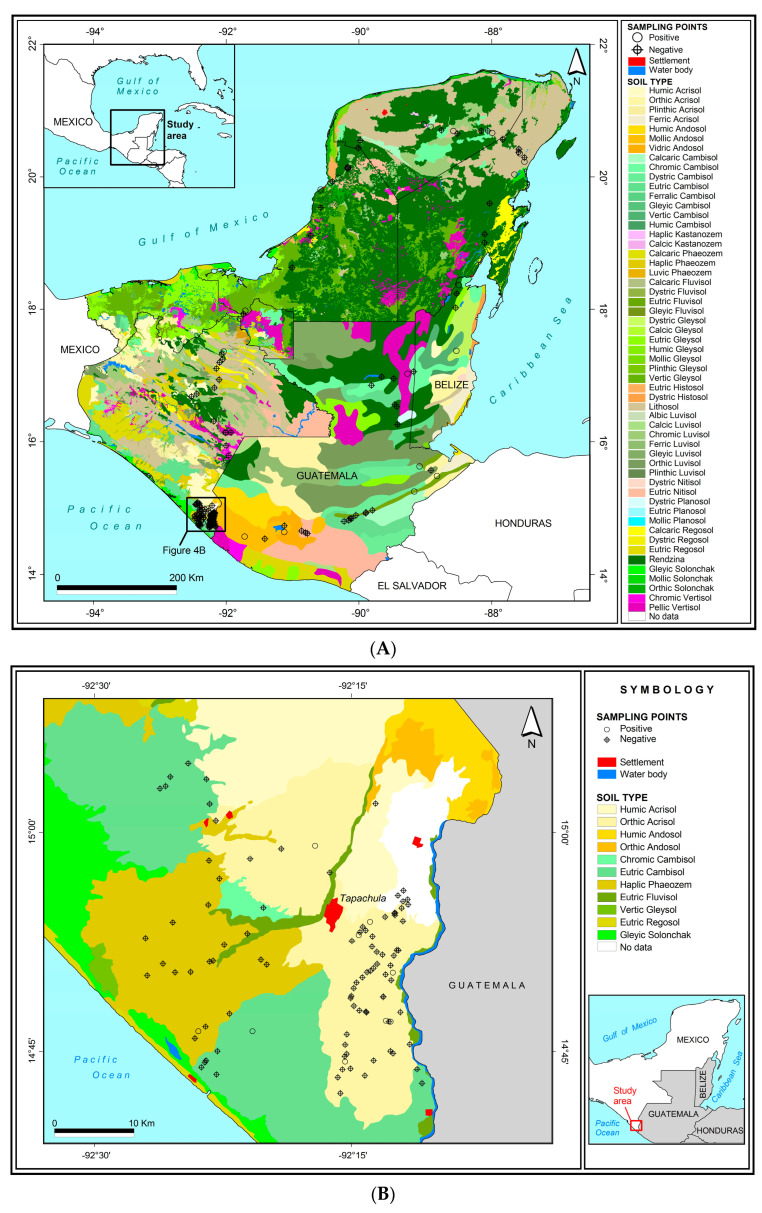
Soil type composite maps for (**A**) southern Mexico, Belize, and Guatemala and (**B**) the Soconusco region of Chiapas State, Mexico. Georeferenced soil samples were plotted as OB-positive samples (open black circles) and OB-negative samples (black circles with a cross). High resolution versions of these maps are available as Supplemental Material (Figures S1 and S2).

Georeferenced information was used to locate samples taken from twelve types of soil, and 16 soil subtypes that are present in this region. The soil type of all but five samples could be identified by plotting sample points on a combined map of the region incorporating data from southern Mexico, Belize, and Guatemala (Figure 4A). The five samples from unknown soil types were classified as “uncharacterized”. Intensive sampling performed in the Soconusco region of Chiapas State, Mexico was plotted on a separate map for clarity (Figure 4B).

**Figure 5:**
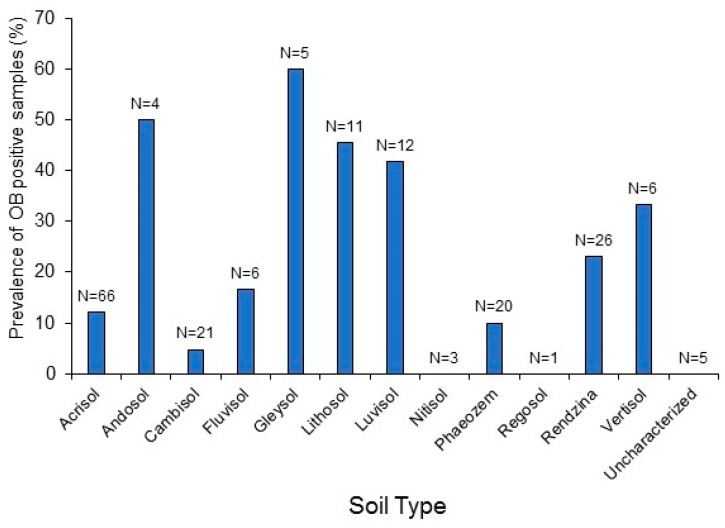
Prevalence of samples from different types of soil that were positive for SfMNPV OBs. Values above columns indicate the number of samples tested (N).

The prevalence of OB-positive samples varied significantly among soil types (*χ*^2^ = 22.998, *df* = 9, *p* = 0.006; soil types with a sample size of <5 were not included in the analysis, namely Andosol, Nitisol, and Regosol). Compared to the average of 18.8% of OB-positive samples (shown in Table 1), Lithosol and Luvisol soils had a clearly higher than average prevalence of OB-positive samples (Figure 5). This was also the case for Andosol, Gleysol, and Vertisol soils (33–60% OB-positive), but the sample sizes were small for those soils (4–6 samples each). In contrast, Cambisol soils were well represented (21 samples) but had a lower than average prevalence of OB-positive samples. The other soil types (Acrisol, Fluvisol, Phaeozem, and Rendzina soils) had intermediate levels of OB-positive samples that were similar to the overall average prevalence (Figure 5). The numbers of OB-positive samples among the soil subtypes are listed in Supplemental Material Table S1, and a brief description of soil characteristics and properties is provided in Table S2.

## 4. Discussion

A 2600 km-long soil sampling trip around southern Mexico, Belize, and Guatemala was followed by intensive sampling in the Soconusco coastal region of the state of Chiapas. Fall armyworm is a major pest of maize in this region, especially during the rainy season (May–October) when this study was conducted. In general, the ecology of OBs in the soil reservoir is poorly understood, which prompted the present study on the prevalence of OBs in maize fields in Mesoamerica.

Overall, almost one in five soil samples (35 out of 186 samples; 18.8%) proved positive for SfMNPV OBs by insect bioassay, indicating that this could be a productive strategy for obtaining novel virus isolates. A selection of 32 isolates all proved positive for PCR amplification of the *sf58* gene, which encodes a *per os* infection factor (PIF9) present in alphabaculoviruses and betabaculoviruses [33,43] (Supplemental Figure S3). This provides additional evidence that the majority of isolates were SfMNPV, although we were unable to test the isolates that had been gifted to other researchers.

The soil bioassay contrasts with the conventional method of collecting large numbers of *S. frugiperda* larvae from infested fields and rearing them in the laboratory to detect natural SfMNPV infections [24,25,26,28]. The soil bioassay approach has the additional benefit that it could be used for isolate prospection during periods when the pest is not present in the crop, or to quantify soil OB persistence over extended periods of time, even when non-host crop plants have been planted as part of a crop rotation cycle.

The prevalence of OB-positive samples varied across the sampled region, but the prevalence of infection in larvae that consumed soil samples was invariably low (average 3%). Calibration of the soil bioassay indicated that the concentrations of OBs in virus-positive maize field samples were likely between 10^4^ and 10^5^ OBs/g soil. This is not an insignificant amount as the 50% lethal concentration of SfMNPV OBs by droplet feeding bioassay is approximately 2 × 10^4^ OBs/mL in second instar larvae of *S. frugiperda* [44].

The estimated concentration of SfMNPV in soil samples in our study (10^4^–10^5^ OBs/g) was very similar to the concentration of OBs reported in other pest–virus pathosystems in Spain [18], Botswana [45], Canada [46], and Connecticut, USA [47], although much higher concentrations (7 × 10^7^ OBs/g) were reported from soil under trees infested by *Hyphantria cunea* in Japan [48]. It is important to note that these studies all used different methodologies to quantify OBs in soil samples.

In the case of the soil-diet bioassay in first instars, the estimated LC_50_ was 1.9 × 10^6^ OBs/g soil, which compared favorably with values derived from soil-diet bioassays on second instars of 1.4 × 10^6^ OBs/g soil [20] and 2.7 × 10^6^ OBs/g soil, albeit with shorter periods (2–4 days) of feeding on the soil–diet mixture [19]. The reason why the LC_50_ values differed by approximately 100-fold between droplet feeding and soil-diet bioassay techniques is likely due to two reasons: (i) the OB-contaminated soil was diluted ten-fold by the addition of the diet prior to the soil-diet bioassay, and (ii) OBs adhere strongly to components of the soil, particularly clay minerals [49], and so may be less readily solubilized in the insect midgut than purified OBs in water.

The soil + diet bioassay technique was successfully applied in a study of natural OB populations in greenhouse crops attacked by the beet armyworm, *Spodoptera exigua* (Lepidoptera: Noctuidae) in southern Spain. Of a total of 267 substrate samples from greenhouses, 34% proved positive for the homologous nucleopolyhedrovirus (SeMNPV) [18]. Following this, a slightly modified soil + diet bioassay technique was used on early instars of *S. frugiperda* to detect OB-positive samples (8% positive) in soils taken from maize fields in northern Mexico [16]. Subsequent analysis indicated that some of these isolates were mixtures of SfMNPV and a granulovirus, suggesting that the insect colony may have harbored a sublethal granulovirus infection [50]. An alternative explanation involving simultaneous acquisition of both types of virus from a single soil sample is unlikely given the low concentration of OBs in soil. Nevertheless, granuloviruses can also persist in soil for extended periods [51,52].

A selection of the SfMNPV isolates from the present study was gifted to A.M. Martínez-Castillo (Universidad Michoacana de San Nicolás de Hidalgo, Mexico), and two of them, from Chiapas and Yucatán, were subjected to analysis of insecticidal characteristics in different Mexican populations of *S. frugiperda*. The Yucatán isolate was also used for field tests with promising results [30], underlining the value of soil OB populations as a source of new and highly insecticidal isolates.

Larvae that became infected after consuming soil samples probably only consumed a single OB, as consumption of one OB was previously demonstrated to result in 2.7–5.4% mortality in *S. frugiperda* second instars [53]. Nonetheless, restriction endonuclease analysis indicated that genetic diversity was present in each of the isolates tested, as evidenced by the presence of fragment size polymorphisms and sub-molar fragments (Figure 4A,B). This was likely due to the presence of genotypic diversity present within individual OBs present in the soil. Restriction endonucleases provide a rapid visual assessment of genetic similarities between isolates but are of limited value for determining the presence of genetic diversity as large fragments, and sub-molar bands may be the result of incomplete digestion of genomic DNA. Nonetheless, we performed several digestions of viral DNA with different enzymes such as BamHI and EcoRI (data not shown), as well as several digestions using HindIII and PstI (shown in Figure 2A,B), and in all cases sub-molar bands were present, which lends support to the idea that the isolates comprised mixtures of genotypic variants.

The SfMNPV-NIC isolate comprises a mixture of nine genotypic variants in various proportions that are enveloped together within ODVs and co-occluded in OBs [53,54]. Genotypic diversity has a marked influence on the transmission and persistence of nucleopolyhedroviruses [54,55,56,57]. A study on OB populations in greenhouse soil substrate revealed that the prevalence of different genotypic variants of SeMNPV varied seasonally and across different parts of the study area, suggesting that certain variants may be better adapted than others to persist outside the host [18]. However, the genotypic diversity of OBs on plant surfaces, in infected insects, and in the soil has not yet been subjected to systematic comparison.

The viable OB population in the soil reflects the result of processes that introduce OBs and those that eliminate OBs from the soil habitat. OBs released from virus-killed insects that fall off the plant and from OB-contaminated foliage are washed onto the soil by rainfall [1] and through the shedding of OB-contaminated leaves [2,48]. As a result, there is a clear correlation between the presence of the pest on the crop and the abundance of OBs present in the soil [18,46,51,58]. This was confirmed in the present study as we detected a positive association between OBs in soil samples and the living stage of the crop cycle, prior to harvest, that is the stage attacked by *S. frugiperda* larvae (Figure 3A,B). Contrarily, processes that eliminate OBs include UV-irradiation and high temperatures at the soil surface, loss through OB transportation onto plant surfaces by wind and rain-splash, and chemical and microbial degradation of OBs in the soil [59,60,61]. In addition, processes such as water percolation, ploughing, the movement of livestock or the activity of the soil fauna can be responsible for altering the vertical distribution of OBs in soil [20,58,61].

An important finding was that the prevalence of OB-positive samples varied significantly among soil types with higher than average prevalence in Lithosol, Luvisol, Andosol, Gleysol, and Vertisol soils, although sample sizes were reduced for the later three soil types. Soils are extraordinarily variable in their physico-chemical and biological properties. However, it is worth noting that Luvisols, Andosols, Gleysols, and Vertisols are characterized by high clay content, which is known to strongly bind to OBs [49]. Surface charge and hydrophobic surface attributes appear to be involved in the interaction of OBs with soil minerals [62,63]. Lithosols are also known as Leptosols and are characterized by very shallow soils over rock, highly calcareous or stony material without clearly expressed horizons. They comprise a wide variety of soils with a diversity of chemical and physical properties. Interestingly, Cambisol soils had a lower than average prevalence of OB-positive samples. Cambisols are known as “brown soils”, that are in the beginning of soil formation and have weak horizon differentiation. Cambisols are characterized by the absence of a layer of accumulated clay, humus, soluble salts, and iron or aluminum oxides [64,65,66,67]. The cause of the low prevalence of OBs in Cambisols may be related to the low clay content or some other factor(s) (see Supplemental Table S2). Finally, although we did not measure soil pH, it is known that pH affects the long-term persistence of OBs in soil both at low pH (acidic) and high pH (alkaline) [51]. Soil pH may also affect surface charge and the tendency of OBs to bind to soil particles [63], which is another field of research that merits systematic examination using modern analytical chemistry techniques.

These findings have intriguing implications in that they suggest that soil type could have a direct effect on the persistence of lepidopteran nucleopolyhedroviruses in the environment. However, on a note of caution, additional factors such as the local maize planting practices, the phenological development of the crop and the density of the *S. frugiperda* infestation could also affect the likelihood of detecting OBs in soil samples, although we did not measure these variables in the present study. It would be interesting to examine systematically the influence of soil type on the prevalence of virus-induced disease in similar pest–crop combinations grown in different types of soil, to explicitly test the hypothesis that soils that favor OB persistence can augment the biological control of lepidopteran pests.

## 5. Conclusions

We conclude that the soil represents an enormous and largely untapped source of novel nucleopolyhedrovirus isolates. Such isolates harbor genotypic diversity that could be useful in the development of biological insecticides. The probability of isolating novel virus strains increased during periods of vegetative growth when the pest was likely present in the maize crop. Certain soil types appeared to be more amenable than others to the persistence of soil OB populations. That soil type could manifestly influence biological pest control by these viruses is a notion that requires systematic evaluation. Finally, the soil-diet bioassay technique has clear potential for the study of the ecology of soil OB populations and the factors that mediate OB persistence in soil, such as soil composition, agronomic practices, and the role of soil invertebrates in OB dispersal.

## Figures and Tables

**Table 1:** Results of insect bioassays on soil samples according to Mexican State and country.

State/Country ^1^	No. of Samples Tested	No. of Positive Samples (%)	No. of Larvae Tested Against OB-Positive Samples	No. of Virus Killed Larvae (%)
Chiapas	120	14 (11.7%)	765	22 (2.9%)
Tabasco	1	0 (0%)	-	-
Campeche	8	4 (50.0%)	240	8 (3.3%)
Yucatán	16	6 (37.5%)	360	15 (4.2%)
Quintana Roo	10	3 (30.0%)	180	8 (4.4%)
Belize	4	2 (50.0%)	120	2 (1.7%)
Guatemala	27	6 (22.2%)	360	6 (1.7%)
Totals:	186	35 (18.8%)	2025	61 (3.0%)

^1^ A single soil sample from Tabasco state was negative in the insect bioassay and was not included in the statistical analyses.

## Data Availability

All the data presented in this study are available in the file Supplementary data file FileS1.xlxs.

## Supplementary Materials

The following supporting information can be downloaded at: https://www.mdpi.com/article/10.3390/insects14010080/s1, (FileS1.xlxs); Figure S1: High-resolution version of Figure 4A; Figure S2: High-resolution version of Figure 4B; Figure S3: PCR amplification of soil isolates using primers targeted at the *sf58* gene of SfMNPV; Table S1: Number of OB-positive samples according to soil type and subtype; Table S2: Properties and characteristics of soil types that differed in the prevalence of OB-positive samples.
