# Supplementary material for: Presence of Spodoptera frugiperda Multiple Nucleopolyhedrovirus (SfMNPV) Occlusion Bodies in Maize Field Soils of Mesoamerica
Source: Insects. 2023 Jan 13;14(1):80. doi: 10.3390/insects14010080 (PMC9864064; doi:10.3390/insects14010080)
Supplement: Supplementary file 1 [file insects-14-00080-s001.zip › Figure S3.pdf]

Figure S3. PCR amplification of soil isolates using primers targeted at the *sf58* gene of SfMNPV

A.

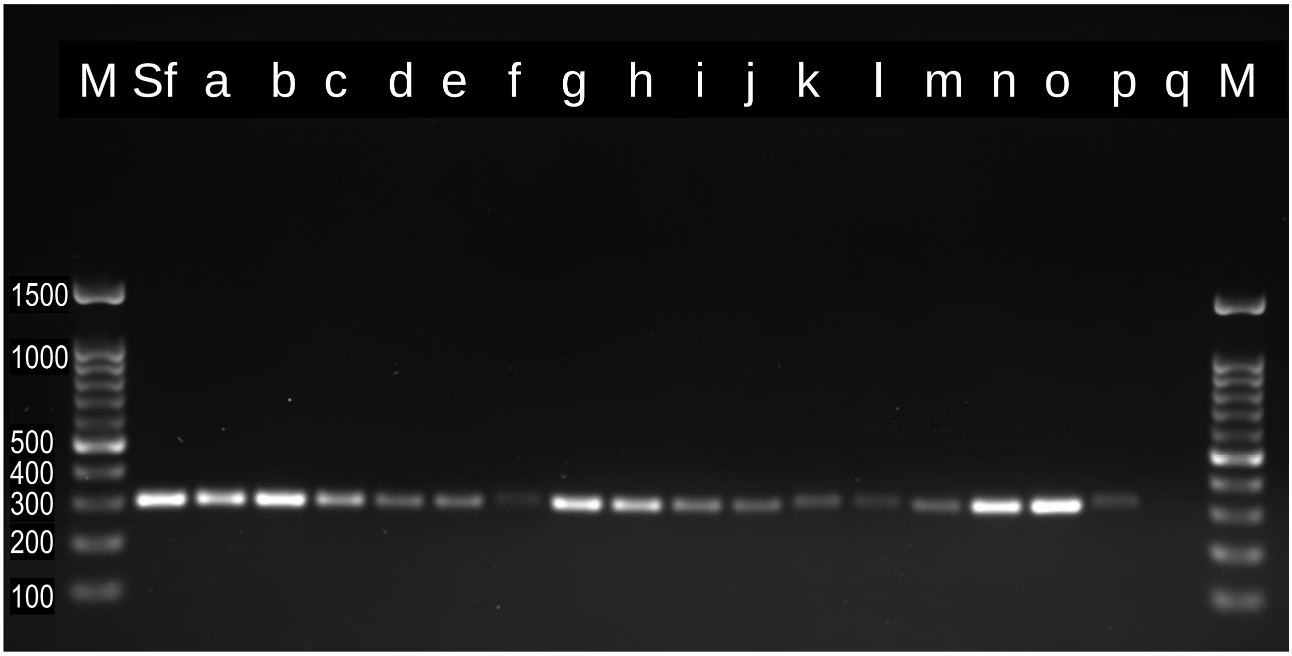

B.

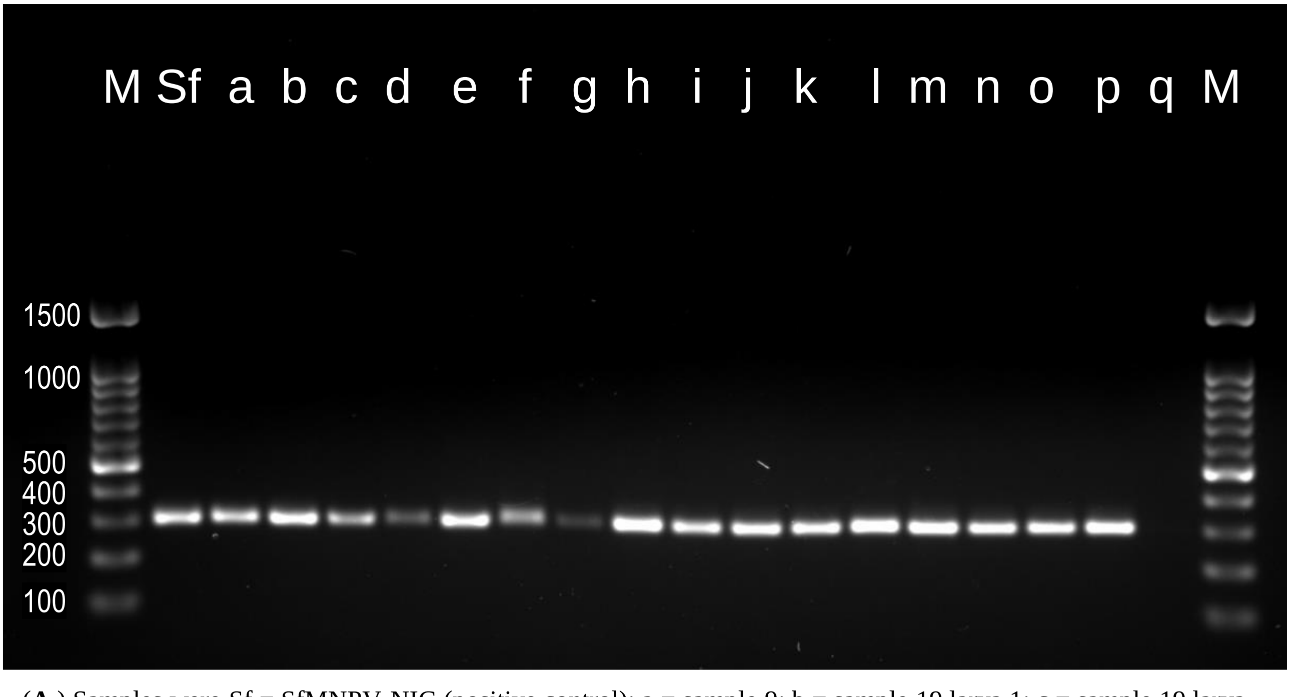

(**A.**) Samples were Sf = SfMNPV-NIC (positive control); a = sample 9; b = sample 19 larva 1; c = sample 19 larva 2; d = sample 21; e = sample 18; f = sample 25 larva 1; g = sample 25 larva 2; h = sample 26; i = sample 28; j = sample 30 larva 1; k = sample 30 larva 2; l = sample 30 larva 3; m = sample 30 larva 4; n = sample 36 larva 1; o = sample 36 larva 2; p = sample 37 larva 1; q = negative control (water). M = 100 bp ladder (GoldBio, St Louis, MO, USA).

(**B.**) Samples were Sf = SfMNPV-NIC (positive control); a = sample 37 larva 2; b = sample 37 larva 3; c = sample 39; d = sample 50 larva 1; e = sample 50 larva 2; f = sample 51 larva 1; g = sample 51 larva 2; h = sample 67; i = sample 90; j = sample 92 larva 1; k = sample 92 larva 2; l = sample 95; m = sample 122; n = sample 130; o = sample 152; p = sample 177; q = negative control (water). M = 100 bp ladder (GoldBio, St Louis, MO, USA).
