# Supplementary material for: Presence of Spodoptera frugiperda Multiple Nucleopolyhedrovirus (SfMNPV) Occlusion Bodies in Maize Field Soils of Mesoamerica
Source: Insects. 2023 Jan 13;14(1):80. doi: 10.3390/insects14010080 (PMC9864064; doi:10.3390/insects14010080)
Supplement: Supplementary file 1 [file insects-14-00080-s001.zip › Table S1.pdf]

Table S1. Number of OB-positive samples according to soil type and subtype

Soil type	Number of OB-positive samples	Number of samples tested	Soil subtypes (no. positive/no. tested)
Acrisol	8	66	Humic 0/2; Orthic 8/64
Andosol	2	4	Mollic 2/4
Cambisol	1	21	Chromic 0/4; Eutric 1/17
Fluvisol	1	6	Eutric 1/6
Gleysol	3	5	Eutric 1/1; Vertic 1/2; Calcic 1/2
Lithosol	5	11	No subtype information
Luvisol	5	12	Chromic 4/10; Orthic 1/2
Nitisol	0	3	Eutric 0/3
Phaeozem	2	20	Calcaric 1/2; Haplic 1/18
Regosol	0	1	Calcaric 0/1
Rendzina	6	26	No subtype information
Vertisol	2	6	Chromic 0/1; Pellic 2/5
Uncharacterized	0	5	
Totals	35	186	
