# Supplementary material for: Presence of Spodoptera frugiperda Multiple Nucleopolyhedrovirus (SfMNPV) Occlusion Bodies in Maize Field Soils of Mesoamerica
Source: Insects. 2023 Jan 13;14(1):80. doi: 10.3390/insects14010080 (PMC9864064; doi:10.3390/insects14010080)
Supplement: Supplementary file 1 [file insects-14-00080-s001.zip › Table S2.pdf]

Table S2. Properties and characteristics of soil types that differed in the prevalence of OB-positive samples.

Soil type	Characteristics and properties
Lithosol	Lithosols are very shallow soils over rock, highly calcareous or stony material without clearly expressed morphological features and with weak horizons. They comprise a wide variety of soils with a diversity of chemical and physical properties. Their dark brown or black calcareous organo-mineral surface soil often has a well-developed crumb or granular structure, or a vermicular structure with abundant earthworm activity. They can be common in mountainous regions where material is rapidly removed by erosion. Most Lithosols harbor sparse shrubs, grassland or forest. They are generally unattractive for agriculture because of their inability to hold water. Lithosols are known as Leptosols in the World Reference Base for Soil Resources (WRB) classification or orthents in the USDA classification. Leptosols on calcareous rock were previously described as Rendzinas by the FAO.
Luvisol	Luvisols are characterized by a surface accumulation of humus overlying an extensively leached layer lacking in clay and iron-bearing minerals. Below this lies a layer of mixed clay accumulation, the 'argic' horizon, with high levels of available nutrient ions. The clay minerals have not been extensively weathered and have high cation exchange capacities and high base saturation. Luvisols typically have a brown or dark brown surface horizon over the greyish brown or red argic horizon. They have a granular or crumb surface structure that is porous and well aerated. Luvisols usually form on flat or gently sloping landscapes in temperate and warm regions. The favorable mineralogy and nutrient content, and the good drainage of these soils make them suitable for a wide range of agricultural uses. They are also known as Grey-Brown Podzolic soils or Alfisols (USDA Soil Taxonomy).
Vertisol	Vertisols have a high content of expansive clay minerals that form deep cracks when dry. Alternate shrinking and swelling when wet causes continuous mixing, often resulting in a deep A horizon and no B horizon. Vertical gilgai (cracks) may be present. Vertisols are common in Mexico and typically form from highly basic rocks, such as basalt. The natural vegetation of vertisols is grassland or subperennial forest. The heavy texture and unstable behavior of the soil makes it difficult for agricultural use.
Andosol	Andosols are generally quite young black soils found in volcanic areas. Andosols are usually defined as soils containing high proportions of volcanic glass and amorphous colloidal materials. The typical Andosol has an AC or ABC profile with a dark Ah-horizon of 20-50 cm thick on top of a brown B or C horizon. The volcanic glass, ferromagnesian minerals, feldspars and quartz components in the silt and sand fractions of Andosols differ between sites. The composition of the clay fraction of Andosols varies with multiple factors including parent material, pH, moisture regime, and soil organic matter. The clay fraction many include a variety of minerals such as kaolinite, gibbsite and various silicates. The average

	organic matter content of the surface horizon is about 8% but some profiles may contain as much as 30% organic matter. The surface horizon is very porous and friable, and has a crumb or granular structure. Most Andosols have excellent internal drainage, are typically very fertile and support intensive agriculture, except where phosphorus is rapidly fixed in some tropical regions. Andosols are known as Andisols in the USDA soil taxonomy system.
Gleysol	A gley is a wetland or meadow soil saturated with groundwater for long enough to develop a characteristic gleyic color pattern. The pattern is essentially made up of reddish, brownish, or yellowish colors at surfaces of soil particles and/or in the upper soil horizons mixed with greyish/blueish colors deeper in the soil. More specifically, Gleysol profiles normally have a spongy litter layer over a dark grey Ah-horizon of heavy clay that changes sharply into a mottled grey or olive Bg-horizon. The soil material is hard when dry and sticky when wet. Gleysols are found in low lying landscapes with shallow groundwater. As wetness is the main limitation on agriculture, Gleysols are often covered with natural wetland vegetation or are used for animal grazing. Gleysols are known as Gleyzems, Inceptisols and Mollisols in the USDA soil taxonomy.
Cambisol	In contrast to the previous soils, the prevalence of OB-positive samples from Cambisols was below average. Cambisols, known as 'brown soil', are in the beginning of soil formation in which horizon differentiation is weak. Cambisols are characterized by the absence of a layer of accumulated clay, humus, soluble salts, or iron/aluminum oxides. Cambisols occur in widely differing environments. They comprise medium and fine-textured materials such as sandy loam or finer, derived from a wide range of rocks. They have good structural stability, a high porosity, good water holding capacity and good internal drainage. They also have reasonable chemical fertility and an active soil fauna. As a result, most of these soils make good agricultural land and are used intensively. The USDA Soil Taxonomy classifies these soils as 'Inceptisols'.

Compared to the overall average (18.8%) of OB-positive samples, Lithosol, Luvisol, Andosol, Gleysol and Vertisol soils had a higher than average prevalence of OB-positive samples, whereas Cambisol soils had a lower than average prevalence of OB-positive samples (Figure 5). Information on soil properties was obtained from online sources [65-67].
