# Supplementary figures and images for: Presence of Spodoptera frugiperda Multiple Nucleopolyhedrovirus (SfMNPV) Occlusion Bodies in Maize Field Soils of Mesoamerica
Source: Insects. 2023 Jan 13;14(1):80. doi: 10.3390/insects14010080 (PMC9864064; doi:10.3390/insects14010080)

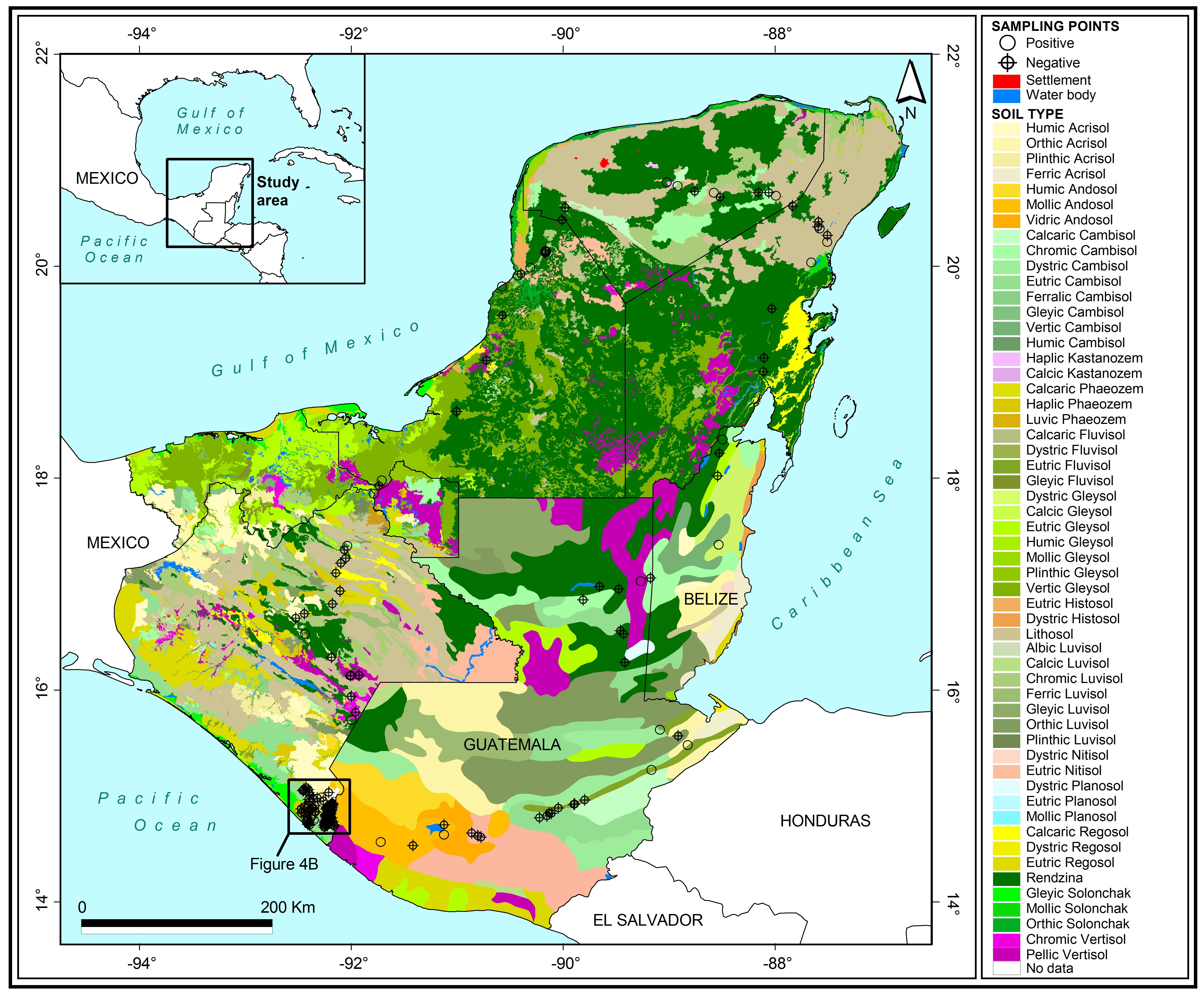

Supplement: Supplementary file 1 [file insects-14-00080-s001.zip › Figure S1.jpg]

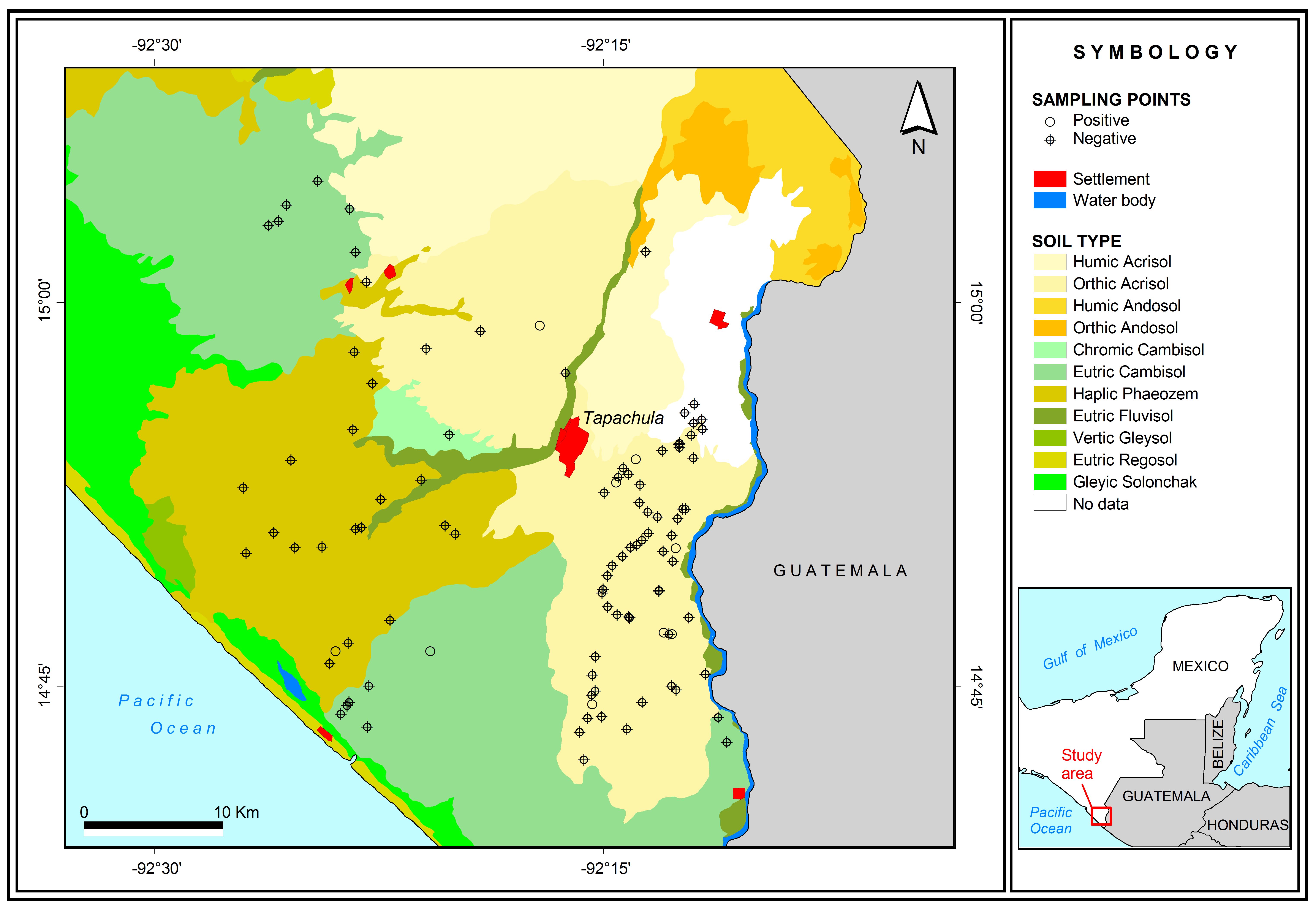

Supplement: Supplementary file 1 [file insects-14-00080-s001.zip › Figure S2.jpg]
